# Computer vision quantization research on the architectural color of Avenida de Almeida Ribeiro in Macau based on the human eye perspective

**DOI:** 10.3389/fncom.2022.951718

**Published:** 2022-09-16

**Authors:** Lina Yan, Qian Li, Yi Zhang, Chun Zhu

**Affiliations:** ^1^Faculty of Humanities and Arts, Macau University of Science and Technology, Taipa, China; ^2^Department of Architecture, School of Civil Engineering and Mechanics, Yanshan University, Qinhuangdao, China; ^3^Department of Architectural Design, Shanghai GOODLINKS International Design Group, Shanghai, China

**Keywords:** computer vision, architectural color, streets of Macao, color identification, quantitative research

## Abstract

In this study, a new quantifiable and refined urban street color analysis method was proposed by combining professional color cards and efficient software color recognition, which solved the problems of low efficiency and difficulty in the quantification of urban color research and analysis. The research mainly uses China Building Color Card (CBCC) and Python (use programs for the HSV color segmentation of pictures) and other software to carry out color recognition for a street view. From the aspects of color composition, type, proportion, visual level, and color sequence of the street facade, this article makes a quantitative analysis of the color of Avenida de Almeida Ribeiro in Macao from multiple angles. The method of combining color card colorimetry with computer color recognition, which not only considers the inherent color of the building but also includes the color situation under the influence of the environment, can express the “actual color situation” of the building more completely. This article quantifies, combs, summarizes, and compares architectural color and environmental color completely. This method has good universality and ease of use in practice, and the conclusion of the study can provide a reference for the color planning of Macao, the color selection of urban renewal has reference significance, and provide a new method for the study of urban color.

## Introduction

### Research background

The urban color shows the unique style and temperament of a city, and streets and buildings are the main manifestations of urban color. With the continuous improvement of the demand for urban space quality, the study of urban color emerged in western countries in the mid-twentieth century. In 1978, French scholar Jean-Philippe Lenclos, established “Atelire 3D Coulour,” which designed and studied the urban color environment of residential and industrial environments in many cities (Lenclos, 2019). In 1996, Professor Michael Lancaster of the University of Greenwich put forward “Color Landscape Theory,” which emphasized: “to show the relationship between colors and colors and between colors and environment, and proposed to show the color characteristics of cities in the context” (Hsiao et al., 2013). Haroldting, a professor of architecture in the United States, took color in architectural design as the object and wrote “Color Consulting” to discuss architectural color in cities (Tang et al., 2020).

In addition to the development of western color theory, Japan is one of the first countries in Asia to study urban color. In the 1970s, based on the research of French scholars, Japan established the Color Planning Center (Hsiao, 1995), an institution dedicated to the study of urban environmental Color. Subsequently, the country such as China, Korea of Italy, Germany, and Asia brings color into urban landscape environment management successively. At the end of 1990, with the introduction of urban color theory in China, urban color received more and more attention in urban construction, and color has been included in detailed urban control planning as a guiding indicator. Nowadays, many cities in China began to put forward representative urban construction colors, such as Beijing proposed compound gray, Harbin proposed beige and white.

Macao not only combines the diversity of Chinese and Western cultures but also shows its unique charm through its urban color. Just as the famous American scholar Jane Jacobs mentioned in her urban diversity theory: diversity is nature to big city (Wickersham, 2001). Urban color is also an objective existence of urban diversity and plays an important role in the urban spatial image. Streets and buildings, as the main embodiment of urban color, do not exist in isolation. The combination of architecture and environment can form a unique urban personality.

### Object of study

Avenida de Almeida Ribeiro in Macao, built-in 1918, is about 580 M long, ending at “Avenida da Praia Grande” (road name) in the east and “Rua das Lorchas” (road name) in the west. The road width is 9–12 M, showing a changing trend of wide in the east and narrow in the west. As a relatively prosperous street in Macao, it has witnessed the development process of Macau's inner port area from the old fishing port wharf to the commercial hotel building. The building near Rua Das Lorchas in the west retains the original overhang structure. In the middle section are the “Instituto Para Os Assuntos Municipais” (Municipal Department) and “Largo Do Senado” (square), a hotel and a bank built-in the twentieth century. The eastern end, near Avenida da Praia Grande, was built after the twentieth century.

Avenida de Almeida Ribeiro is popularly known by residents as “The New Road.” Different from other roads in the region, Avenida de Almeida Ribeiro, a major urban renewal project of Macao in the twentieth century, is an important passageway through the entire inner harbor from east to west based on the original inner harbor area of Macao. Before 1918, Avenida de Almeida Ribeiro was the section from Largo do Senado to the inner-Harbor area. In 1918, after the renovation, the government renamed the new road as “Avenida de Almeida Ribeiro.”

**Figure 1:**
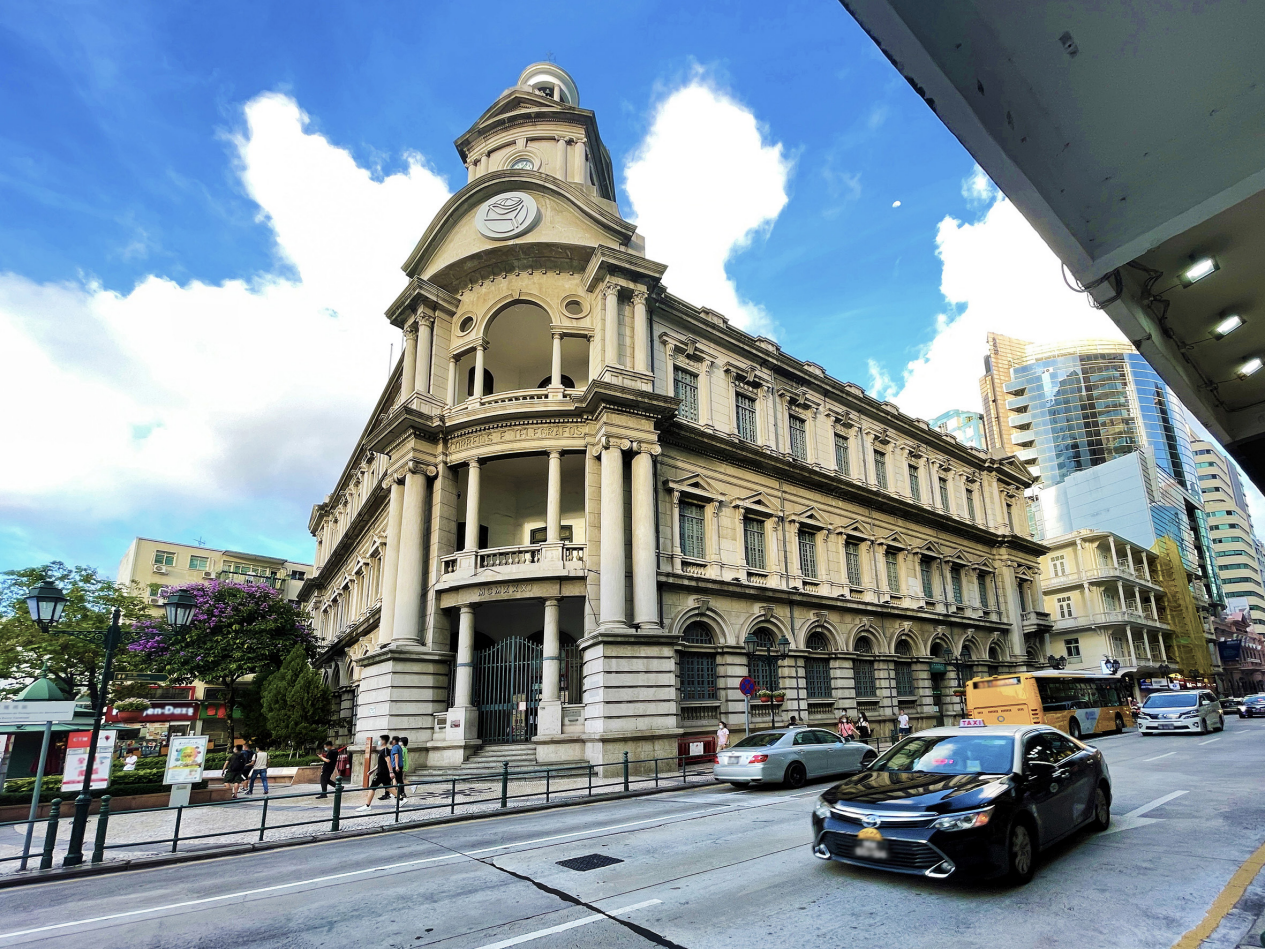
The post office of the Macao special administrative.

**Figure 2:**
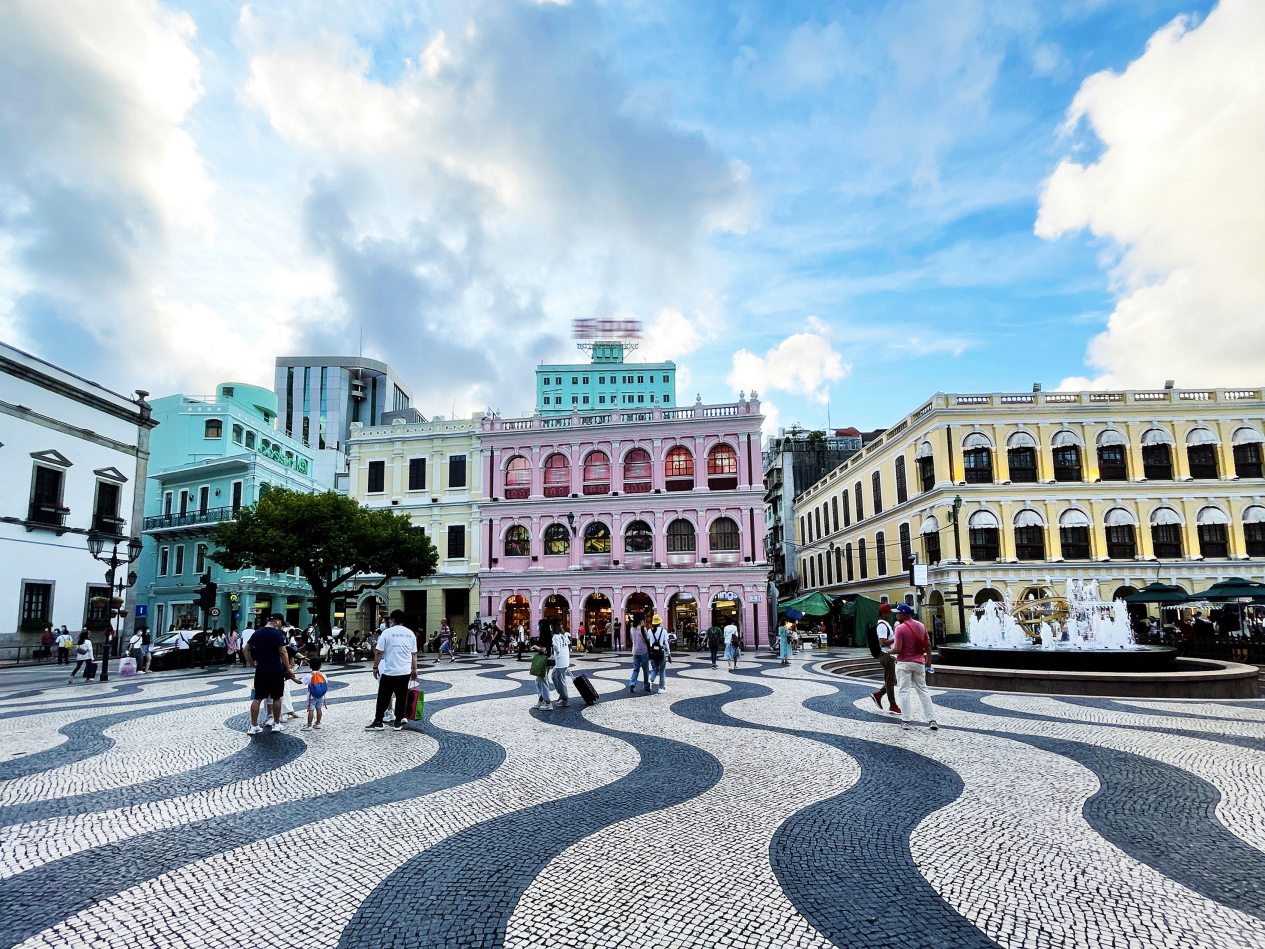
Largo do Senado.

Macao's government attaches great importance to the protection and management of historical and cultural heritage. Avenida de Almeida Ribeiro is an important part of Macao's World Cultural Heritage, and its street facade has retained its original appearance in the early twentieth century. The former municipal and commercial space forms not only show the unique charm of the coexistence of Chinese and Western cultures but also completely retain the original color of the street facade, which is beneficial to the study of the traditional color of Macao city streets in this article (Figures 1, 2).

### Computational vision

Human vision mainly relies on light-sensitive cells in the retina of the eye, and color vision with the cone cells in the retina, the layer of nerve cells that transmits visual signals to the brain. In other words, color perception is based on cells and is a subjective feeling. However, the computer is based on image pixel color data statistics and can be more objective and quantified statistics.

The concept of computer vision was first proposed in 1970 (Szeliski, 2010). It is a method of translating three-dimensional objects into two-dimensional images into pixel numbers, color values, and other information for analysis through a software editing algorithm (Lee et al., 2015). The purpose is to establish a quantitative understanding of spatial images with the help of computers. Compared with the traditional way of using color cards directly or using an electronic color spectrometer to identify the color of buildings, the method of computer vision extraction is more similar and efficient to human visual perception. The traditional color card colorimetric method, through the naked eye judgment, will inevitably produce color perception error. The same color in different environments will be affected by weather, ambient light and other environmental factors and change its original color.

Consequently, this article uses computer vision to quantify color value recognition to make up for the color deviation caused by eye color recognition. Through a picture color segmentation program to obtain a variety of color statistics. This is a more objective and reproducible approach.

## Color extraction and analysis methods

### Foundation of architectural color system

Based on the “Munsell Color System,” the Chinese architectural color system has formed The color standard of GB/T 15608-2006 (The Chinese Color System). The standard divides color into hue (H), value (V), and chroma (C) based on the three attributes of color perception. “CBCC China Building Color Card” was compiled under this standard. This study follows China's architectural color standards, based on the Munsell Color System, with the help of the “CBCC Chinese Architectural Color Card” as the reference for building inherent color sampling, combined with computer vision. “HSV color space” (Sural et al., 2002) (hue—H, saturation—S, and value—V) was used as the extraction of environment color and space color.

The computer algorithm can be easily used in HSV color space to present the hue, saturation, value, and shade of the color in the form of data. The description of HSV color space is close to the human perception of color. HSV encapsulates information about color in ways that are more familiar to humans: “What color is this? What about the depth? How about light and shade?” In addition, these color data can be separately and independently processed to facilitate more refined color quantization research.

### Street color extraction—A combination of old and new methods

The method of this study combines color cards with computer color recognition.

First, “CBCC China Building Color Card” was used to compare the actual buildings with Color cards on-site. To avoid the color difference caused by weather, light changes and environmental reasons, the on-site color taking time is from 9 a.m. to 11 a.m. or from 3 p.m. to 5 p.m. on cloudy days for color card comparison and shooting (18/05/2021–20/05/2021). The number of recorded photographs shall be at least five for each building. We took 460 photos to provide a field data source for subsequent computer color recognition.

Second, color segmentation and recognition of architectural environment color and space color were carried out with the help of Python (a program for the HSV color segmentation of pictures) (Srane96, 2019). This python program (HSV-Color-Range-Calculator) can be used to calculate HSV color ranges for each color and see the result live. We referenced and modified his program for image color processing.

**Figure 3:**
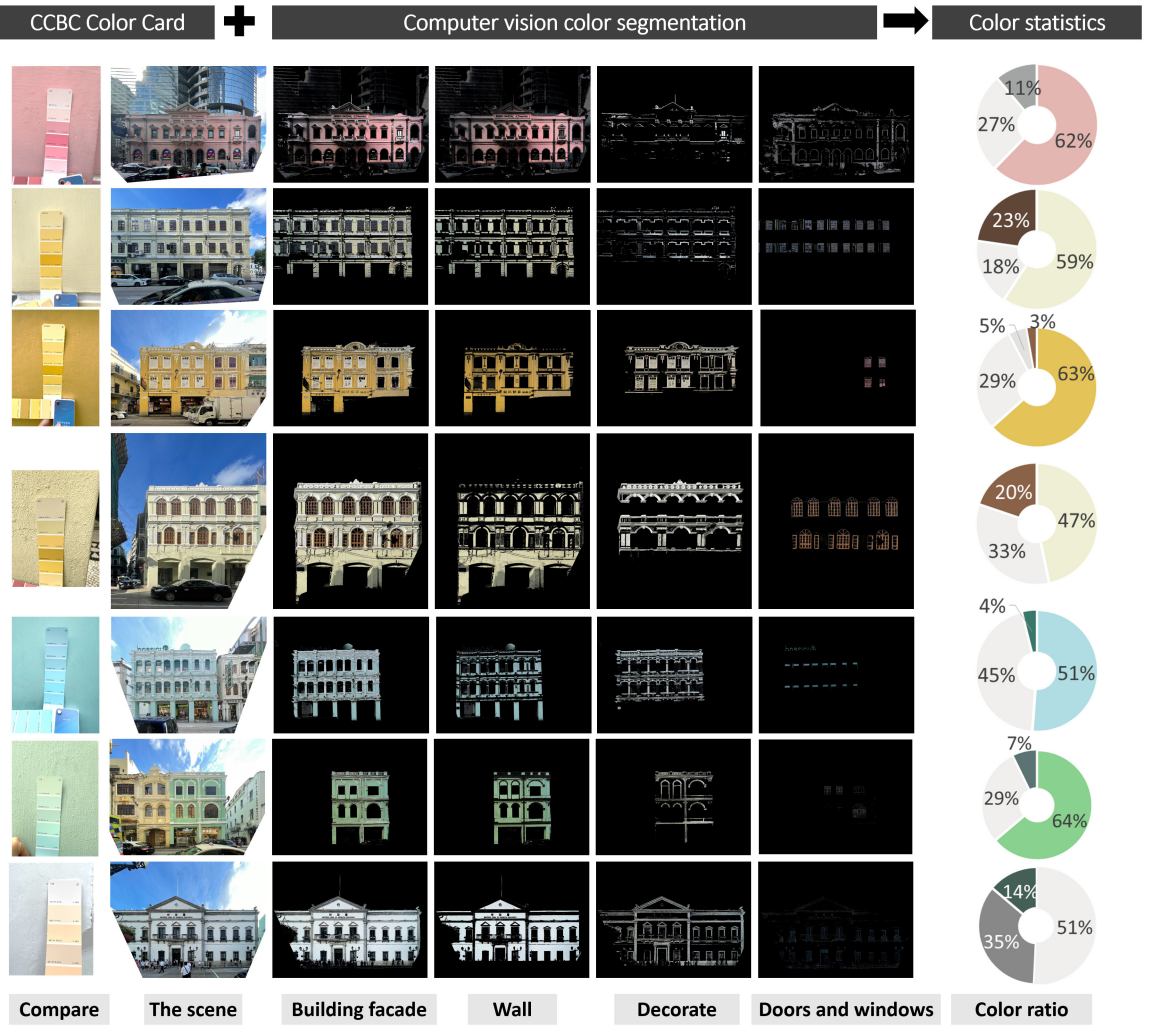
Color card selection and computer vision recognition of architectural color analysis example.

Finally, the obtained color data are the approximate value of the color deviation perceived by human eyes. After sorting out the data, the overall color situation of street building facades can be obtained (Figure 3).

### Color analysis method—Quantitative statistics

After extracting the “inherent color” of buildings mentioned above, the inherent color is classified into main color, auxiliary color, and ornament color according to the ratio of architectural color area and its proportion is counted.

At the same time, the computer is used to perceive and recognize the “environment color” of the building. The color sequence of the whole street building is compared and summarized according to the style characteristics and color types of the building facades on both sides of the street.

Then, with the help of Rhino and Grasshopper and other software combined with the current photos, the influence of distance and color on the “space color” of the building was analyzed.

In general, a relatively complete architectural color classification and analysis system is formed in this article from the three perspectives of “color type, color sequence, and color visual level” of street building facades.

## Analysis of architectural color

### Classification of architectural colors

**Figure 4:**
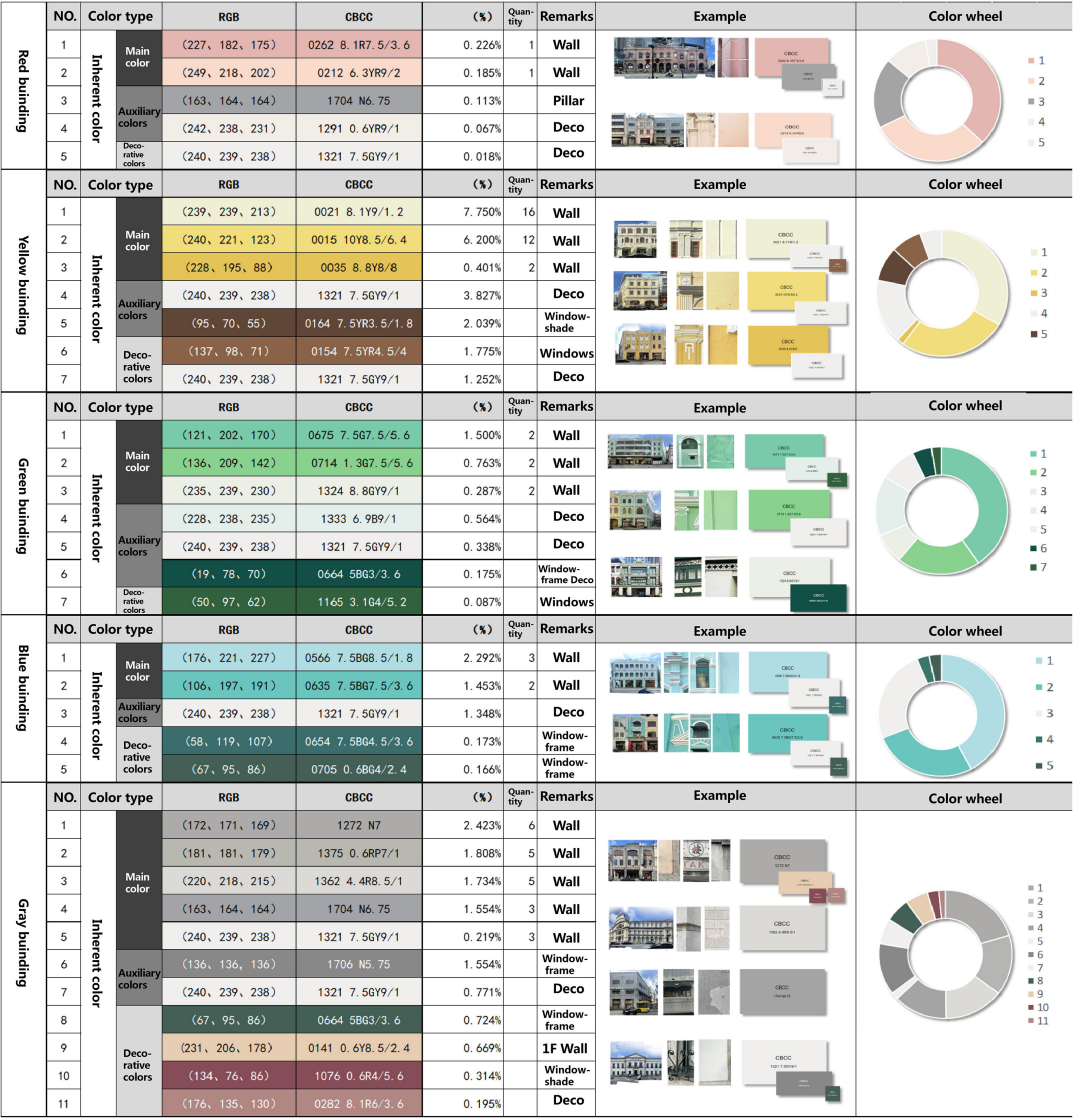
The statistical table of “inherent color” of buildings based on the CCBC color card.

There are a total of 73 buildings on both sides. Using CBCC color cards to compare the walls, doors, windows, and decoration of the buildings, 186 types of color samples were obtained. But many of the colors are the same, so we excluded the same color samples and ended up with 35 colors comparable to the color card. Among them, gray is the most abundant architectural color type and has more decorative colors. The rest is composed of a large wall face and white decoration. From the perspective of inherent color, the main color of street architecture is clear, which can be divided into red, yellow, green, blue, and gray. Auxiliary color is mainly located in the building decoration part of the gray color system. Ornament color is mainly located in the blinds, window frames, and shop sign text position (Figure 4).

Next, this study splices a large number of building facade photos taken by field research into a complete street facade map. As shown in the figure, the upper part is the full elevation of the east side of the street, and the lower part is the full elevation of the west side of the street. The color calculation range is only for the building part, and the sky and ground are not included in the calculation. After stitching, the image size is 319.28^*^71.79 cm, 37,710^*^8,479 pixels, and 300 dpi.

**Figure 5:**
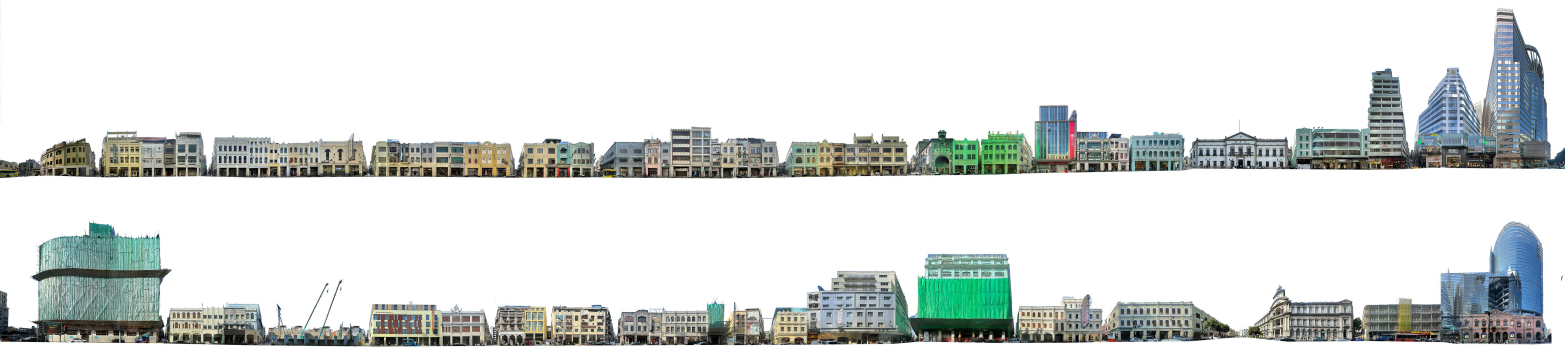
Two facades of Avenida de Almeida Ribeiro, Macau.

On this basis, the “environmental color” of street buildings is recognized by Python. Finally, color data obtained by computer color recognition are quantified and integrated with the inherent color obtained above. Complete quantitative results of street color obtained after statistics are as follows (Figure 5).

In terms of the proportion of color types of the whole building, the number of buildings identified as yellow is the largest, accounting for 33.07%. This was followed by 31.43% gray, 17.15% green, and 15.89% blue red buildings are less, accounting for 2.79%.

From the distribution of the overall building color (Figure 5: H-information) on both sides of the street, yellow, green, and blue occupy the majority, and red is less. It is worth noting that most of the peripheral green and blue colors in polar coordinates are not inherent colors of the building body. Among them, green (10GY ~ 7.5 g) is greatly affected by the construction enclosure with higher purity, and blue (2.5B−10B) is greatly affected by the reflection of the sky. Therefore, excluding these two colors with greater interference, yellow (5RY−2.5Y) can be obtained as the main hue of the street building facade. Followed by yellow-green (10Y−10GY), blue-green (7.5G−2.5B), and red (10RP−7.5R), among which red (near 2.5R) with high saturation is the color of the shop's sign.

**Figure 6:**
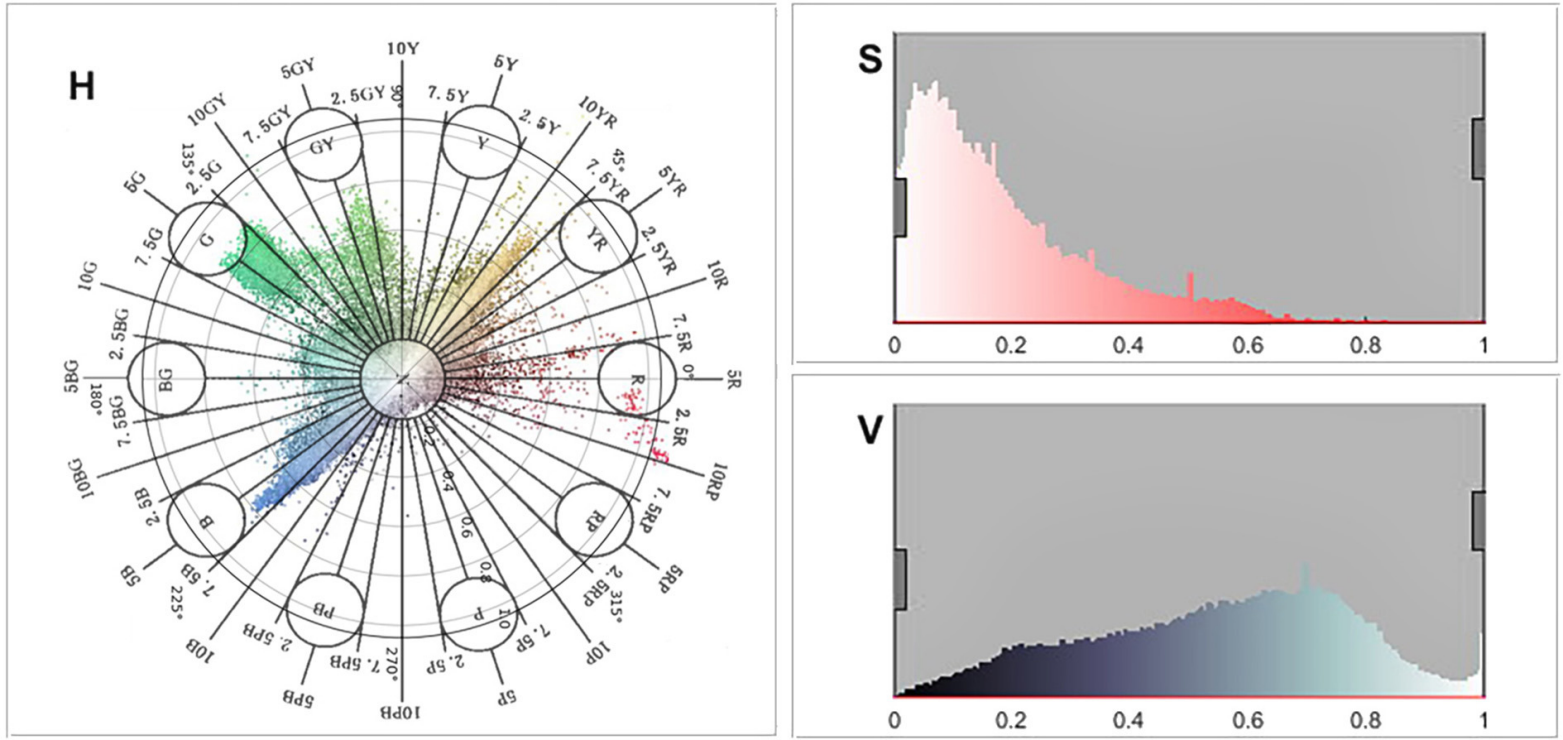
HSV color information analysis diagram.

From the perspective of the overall building color saturation (Figure 6: S-information), the saturation is at a low value, and the overall color saturation of the street is mostly between 0 and 0.6. The overall architectural color lightness (Figure 6: V-information) tends to be medium-high, mostly between 0.2 and 0.8.

**Figure 7:**
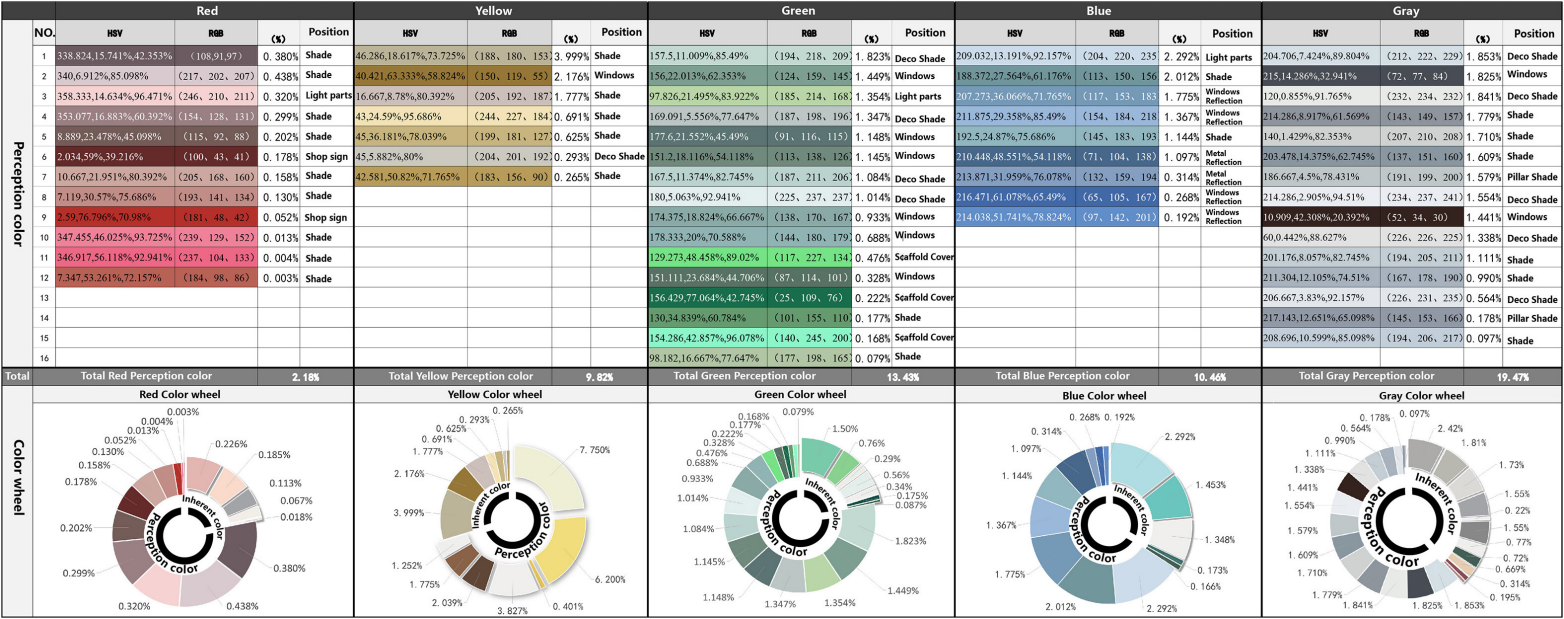
Statistical table of building “environmental color” based on computer vision analysis.

On the whole, architectural colors are characterized by clear hue types, low overall saturation, and medium-high lightness (Figure 7).

### Color sequence of street building facades

**Figure 8:**
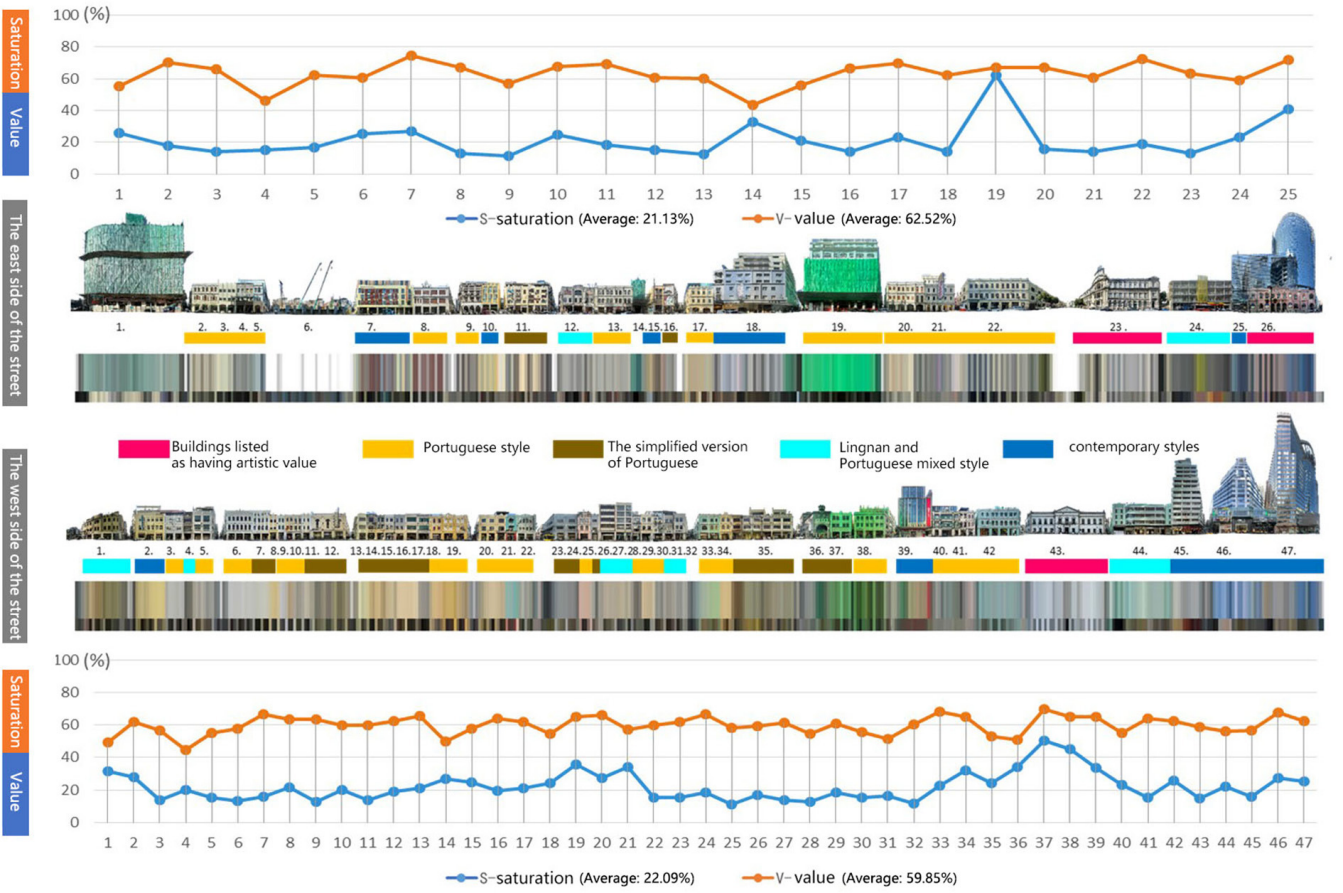
Analysis of the changing trend of building type and facade color lightness and saturation.

As the street with the largest concentration of historical buildings in Macao, Avenida de Almeida Ribeiro's architectural color sequence can better reflect the color characteristics of historical buildings in Macao. According to the location of buildings on both sides of the street, the corresponding lightness, saturation, main color, and auxiliary color of each identified building are arranged and expanded accordingly, that is, the continuous color sequence of the building facade is obtained (Figure 8).

In terms of the value and saturation of the color sequence, the data fluctuation of the west facade is small and the color continuity is good. The lightness and saturation values on both sides of the street are very similar. The average saturation of buildings on the east side is 21.13%, and the average lightness is 62.52%. On the west side, the average saturation is 22.09% and the average lightness is 59.85%. The east side of the building is affected by facade maintenance and construction, the continuity of color is blocked and broken, and there is a high saturation of construction envelope color. Nevertheless, although the continuity of color on the east side is blocked, the intensity and area of maintenance also reflect that Macao attaches great importance to the protection and maintenance of historic building facades.

The style of the architectural is corresponding to the color. The relation between architectural style and color can be obtained by calculating pixel values of different colors. The architectural styles on both sides of the street can be roughly divided into (1) buildings listed as having artistic value; (2) Portuguese-style architecture, (3) the simplified version of Portuguese architecture, (4) Lingnan and Portuguese mixed style architecture, and (5) Contemporary architectural styles. In terms of the color pixel value of each type, Portuguese architecture occupies the highest proportion (42.3%). Portuguese architecture accounted for 30.6% of the total pixels, and simplified Portuguese architecture accounted for 11.6% of the total pixels. Contemporary architectural style occupies the second place, accounting for 35.6% due to its higher overall height and larger color area. Again, 8.7% of Macau's buildings were listed as artistic, including the baroque civil affairs office (Instituto Para Os Assuntos Municipais). The post office of the Macao special administrative, and the preserved pink facades on the ground floor of the Banco Nacional Ultramarino (B.N.U Building). Finally, construction and maintenance accounted for 2.2% of the total. In addition, through the perception of cold and warm architectural colors, it is found that Portuguese classical historical buildings are mainly yellow-green warm colors. Buildings in the Chinese Lingnan style are mainly gray; contemporary buildings are a cool shade of blue with glass walls and marble veneers.

In conclusion, the building color value of the whole street is higher, and the saturation is lower. Higher color value makes the streets look brighter, and lower saturation makes the street feel softer. These styles not only blend on the same road but also maintain their color characteristics and coordinate with each other, forming the color gene barcode with the characteristics of Macao City.

### Visual hierarchy of street architectural colors

#### Define the visual hierarchy of colors

Based on human field angle, with the help of Python and GH (Grasshopper parametric analysis software), the spatial color recognition of street buildings affected by distance is studied, that is, the color change analysis of street buildings located in the front, middle, and back of different visual levels.

The computer color perception setting is based on the height of the human eye position. From the average sizes of men and women in China (male: 1.75 m, female: 1.63 m), the total average height is 1.69 m (World Data, 2020). Some studies have demonstrated that the ratio of head height to body height in adults is 1:7.5 and the height range of the eyes is 1/2 of head height (Shi and Huang, 2015). Therefore, according to the calculation, the average eye height of Chinese adults is about 1.58 m.

**Figure 9:**
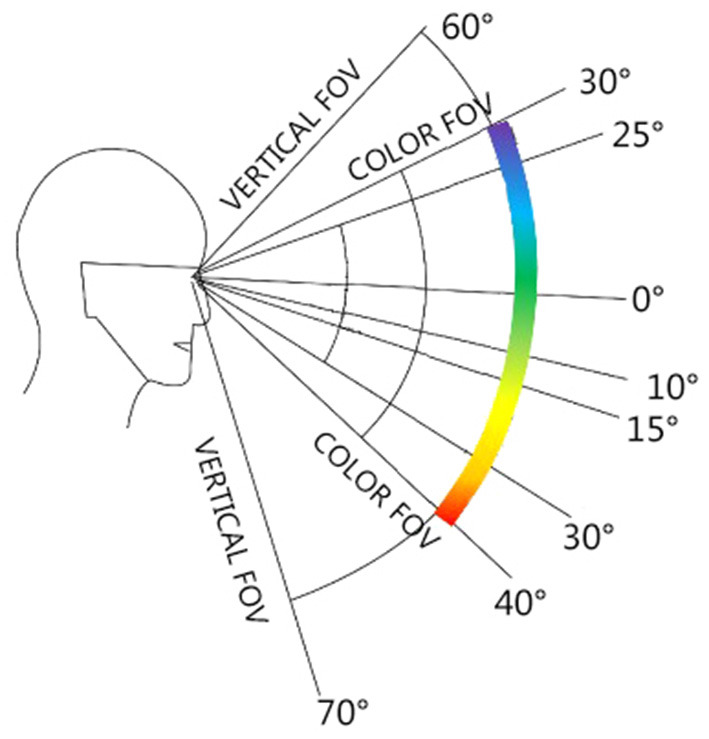
The range of color discrimination of human visual field (vertical direction).

**Figure 10:**
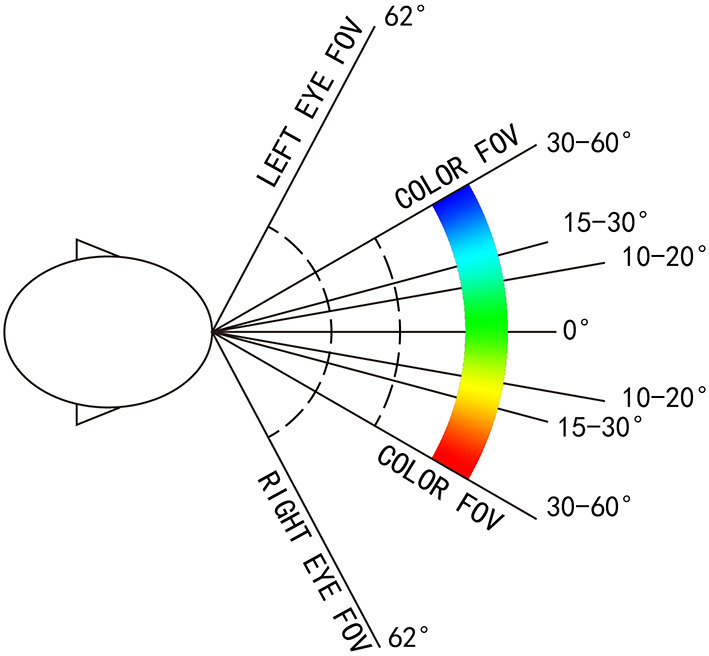
The range of color discrimination of human visual field (horizontal direction).

The upper and lower limits of the visual field color discrimination range are 30° up and 40° down. The left and right boundaries are 30°-60° to the left and 30°-50° to the right (Mollon, 1982) (Figures 9, 10).

In addition, the perceived depth of the view level is divided into three scales according to “Exterior Modular theory,”(Ashihara, 1981) which is convenient for calculation and statistics. The observation range is set as follows: 0–25 m is the close-up view that can see the details of the building; The mid-range from 25 to 100M of the building outline can be observed; able to see objects with the blurred outline of 100M or more in distance by color or light (Yang et al., 2020).

To have a comprehensive understanding of people's different visual feelings in the two directions of the street, six isometric observation sections were set between the beginning and end of the sidewalk on both sides of the street with a total length of about 590 M according to the 100 M boundary of the vision. There were seven observation points including the starting and ending points, and each observation point was a two-way forward and backward observation. Thus, a total of 14 observation angles were used to analyze the spatial color of street building facades.

#### Visual hierarchy analysis of color

According to the overall color level perception analysis of street building facades, the spatial color of the street building facade is affected by distance, street width, and building height.

**Figure 11:**
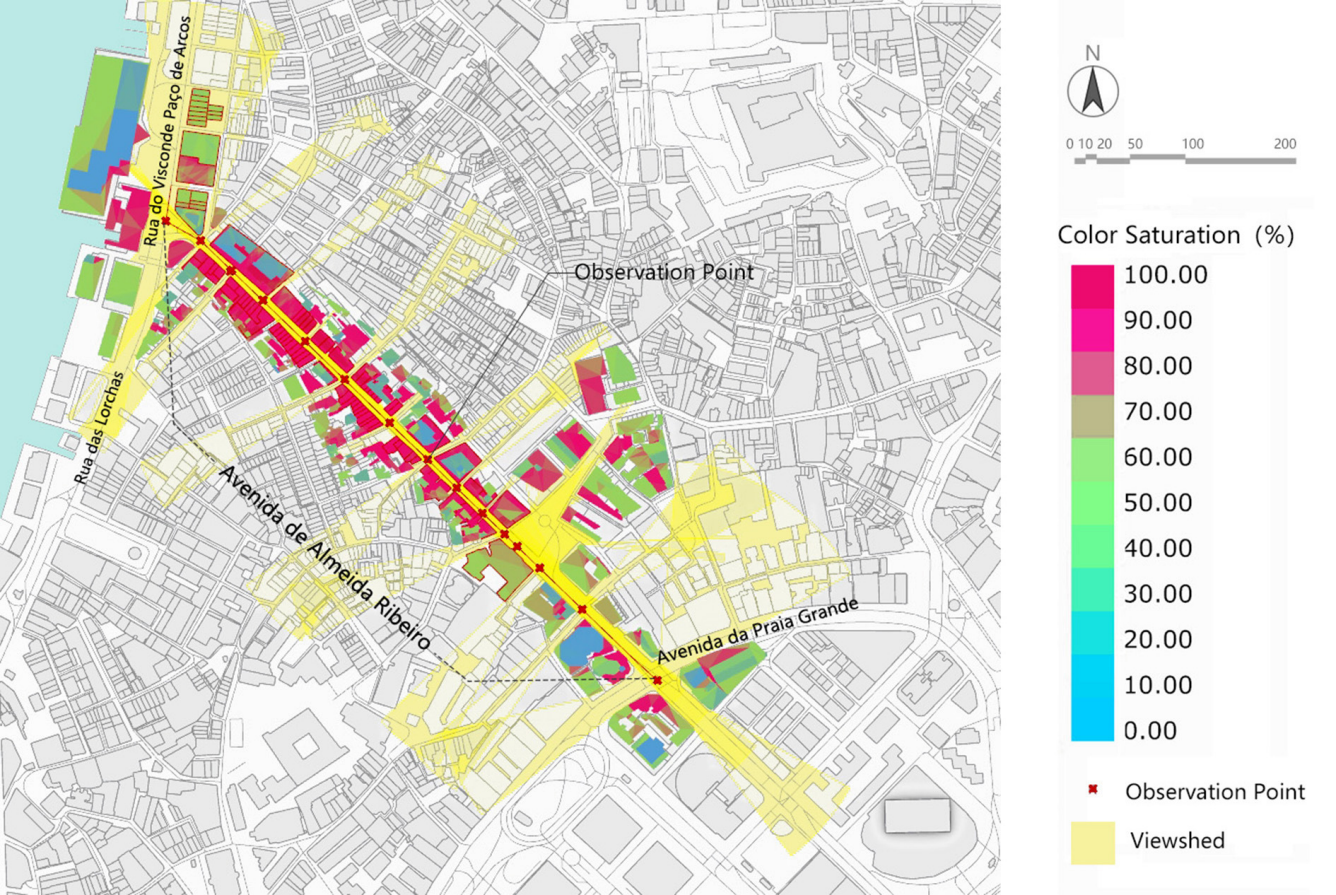
Analysis of Architectural Color Hierarchy perception of Avenida de Almeida Ribeiro in Macao.

Avenida de Almeida Ribeiro is 9–12 M wide. The road is wide on the east and narrow on the west. The horizontal angle of view is set to 0–25 m at close range, which can completely cover the color recognition of the building facades on both sides of the street. For the vertical view, the limits are set to 30° up and 40° down. Avenida da Praia Grande to Largo Do Senado is dominated by high-rise buildings. The section from Largo do Senado to Rua do Visconde Paco de Arcos is dominated by the three stories Macau Varanda building with a height of about 15 M. Therefore, the spatial color perception of the section of high-rise buildings is dominated by the color of low-rise buildings. According to the calculation, the color perception degree of the high-rise building section is 40–70%; the section of the Macau Varanda building can completely cover the building facade, and the color perception is 80–100%. The overall color perception degree is bounded by Largo do Senado, presenting two obvious spatial color perception states (Figure 11).

Thus, it can be seen that the integrity of spatial color perception can be better ensured by controlling the width of the street and building height within the close-range view of 25 M.

**Figure 12:**
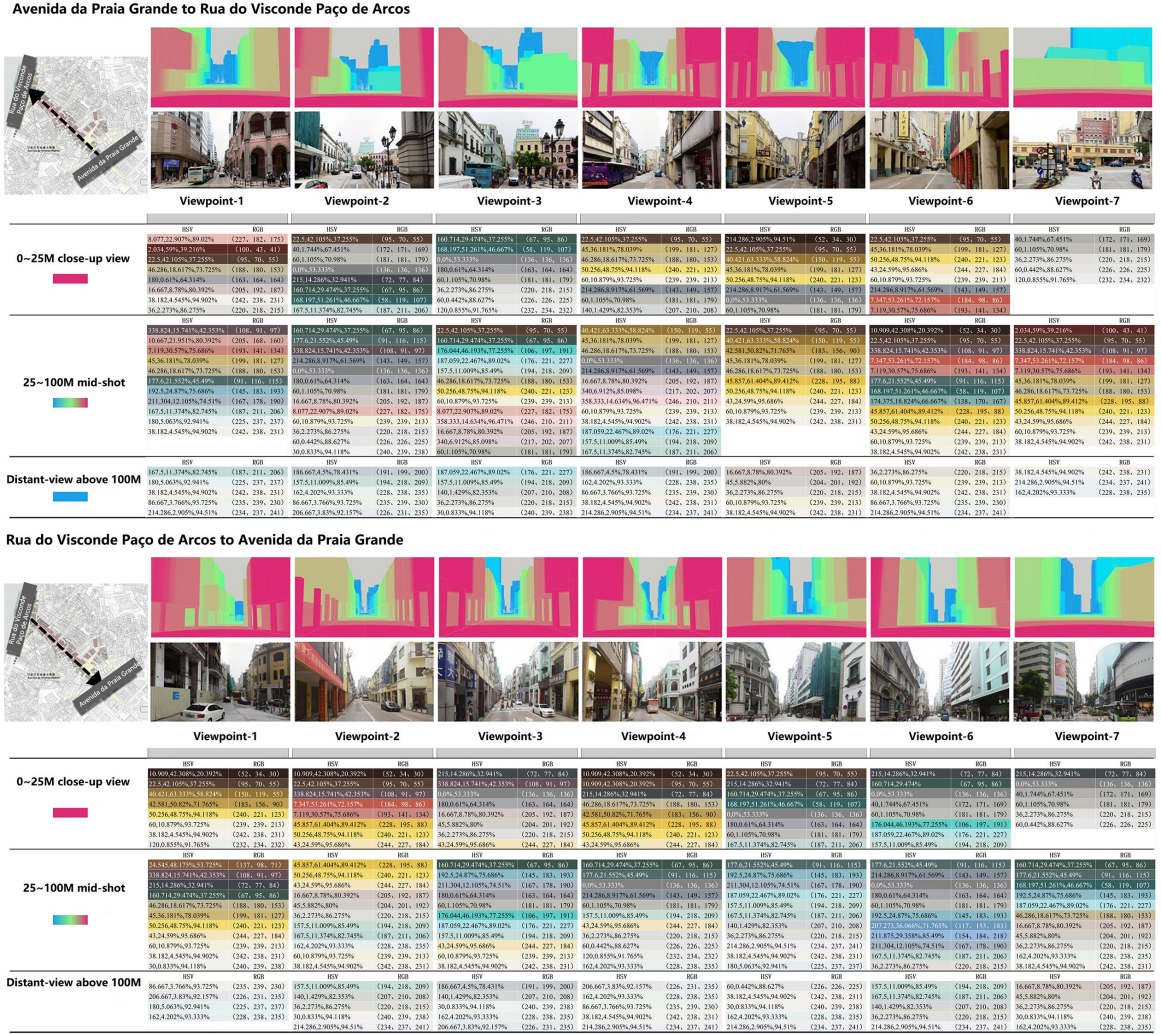
Statistical table of spatial color levels and colors of 14 observation points in two directions of the street.

At the same time, space color is greatly affected by the distance between the two directions of the street. Avenida de Almeida Ribeiro is 590 M long and has a relatively straight road, which is relatively transparent from one end without large buses. This kind of road state has a great influence on the architectural space color on both sides of the street. As the depth of field changes, the greater the refraction of light by atmospheric dust at greater distances, the less clear the distant buildings become. It also reduces color saturation. In good weather, the dust refracts atmospheric blue light more, and the farther away from the building, the bluer it is. The color and the distance of the building present a subtle hierarchical change (Figure 12).

**Figure 13:**
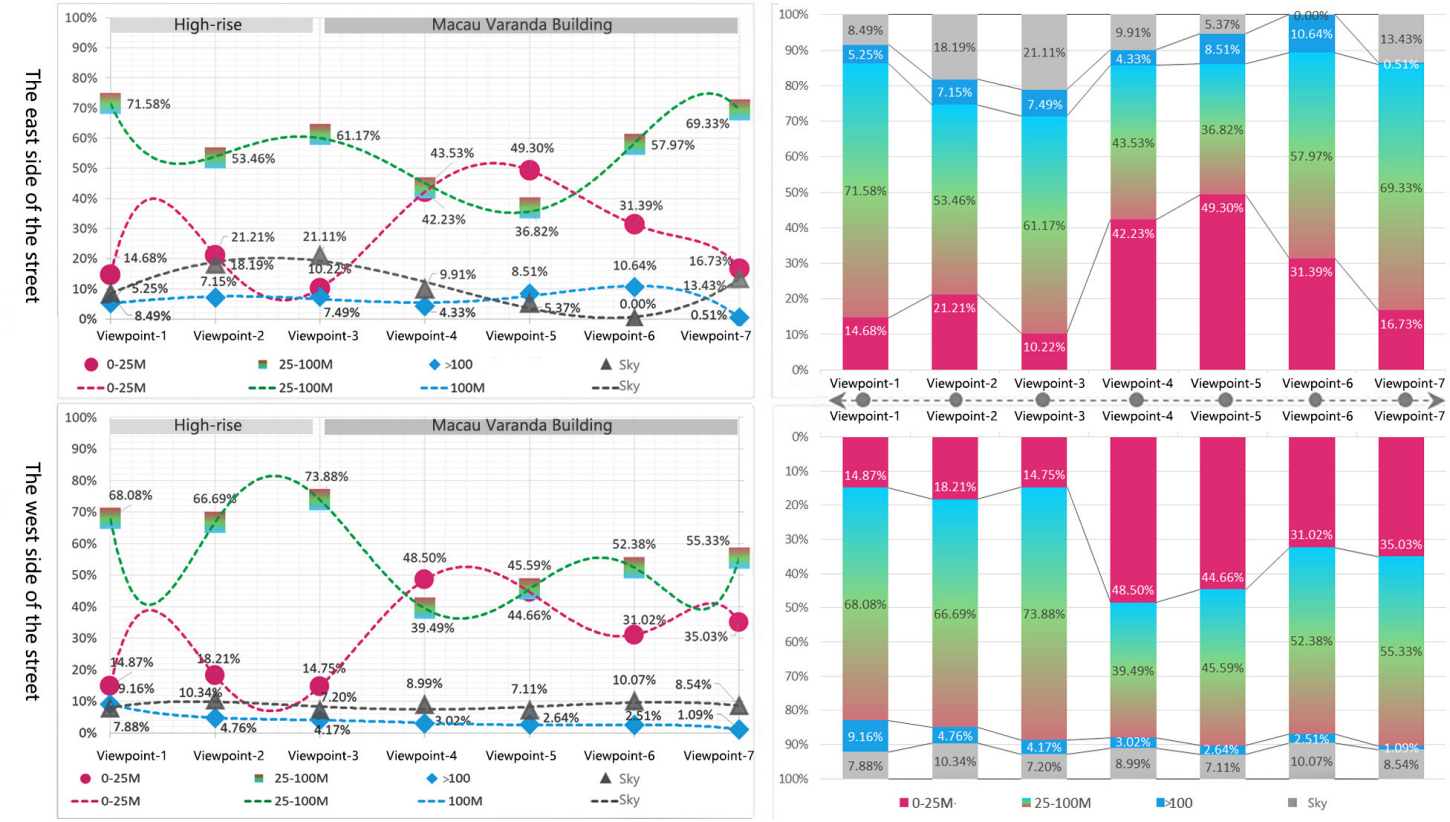
Space color perception area and trend of observation points.

From the data of the specific spatial color perceptive area, the variation trend of 0–25 m close-range in both directions is large, and the value is in the range of 10.22–49.30%. The variation of mid-shot from 25 to 100 M ranged from 36.82 to 73.88%. Distant-view above 100 M has little variation, ranging from 0.51 to 10.64%. In addition, according to the data changes in observation points, it can be found that Largo do Senado (View point-3) is also the cut-off point, and there are two obvious trends of change: The “high-rise section” between Avenida da Praia Grande and Largo do Senado has a relatively small close-range area, ranging from 10.22 to 21.21%, and its color is mainly blue and gray. The area of the mid-shot is generally larger, with values ranging from 53.64 to 73.88%. The value and saturation of colors are relatively high, especially in the vicinity of observation point 3 (Figure 13).

While the close view of the “Macau Varanda Building Section” from Largo Do Senado to Rua do Visconde Paco de Arcos is more than that of the “High-rise Section,” the close-range value is between 16.73 and 49.30%, and the color is mainly yellow and gray. The mid-shot is less than the “tall building section,” the value is between 36.87 and 69.33%, and the color is mainly yellow-green. From another point of view, the spatial color perceptive area of the “High-rise section” is characterized by less close-range and great mid-shots, while the proportion of close-up views and mid-shots of the “Macau Varanda Building Section” is large and fluctuates steadily.

In addition, as the space color perception of “Distant-view” is affected by the height of buildings at both ends of the road, the spatial color perception shows different rules. Avenida de Almeida Ribeiro is surrounded by tall buildings at both ends, so the spatial color area of the “Distant-view” is affected by the height of distant tall buildings both in the forward and backward directions. The range of “Distant-view” in two directions was 0.51–10.64% and 1.09–9.16%, respectively.

The data in both directions have their characteristics. According to distant-view spatial color statistics from Avenida da Praia Grande to Rua do Visconde Paco de Arcos, except for the endpoint of Rua do Visconde Paco de Arcos, the area of the distant-view in this direction remains between 5.25 and 10.64%. As the endpoint of this direction is a T-junction and the buildings at the end are in large yellow tones, the distant-view is almost blocked by the tall buildings on the opposite side of the road, and the color perception level of the view space is mainly yellow in close-up view and mid-shot. Although the blue-gray color of distant-view is only 0.51%, it continues the yellow color characteristics of this section of the Macau Varanda building.

From Rua do Visconde Paco de Arcos to Avenida da Praia Grande, the trend is obvious, showing a gradually increasing trend from 1.09 to 9.16%. This is because Avenida da Praia Grande is directly connected to Avenida do Infante D. Henrique (road name) with the same long value, which further extends the distant space. Avenida do Infante D. Henrique is dominated by tawdry high-rise commercial buildings and hotels. The sense of space of color spreads from blue-gray in close-up views to yellow-gray of distant-view.

## Conclusion

This article takes the color of the Avenida de Almeida Ribeiro in Macao as the research object and analyzes the color classification from the three aspects of “inherent color,” “environment color,” and “space color” of the street facade. After extracting the “inherent color” from the CBCC Chinese architecture color card and quantifying the “environmental color” by the Python program, the phenomenon and rules of color classification research on the facade of the Macau World Cultural Heritage street building are discussed in terms of color types, color sequences, and color levels. Thus, a multi-angle street building facade color research system is formed, which can comprehensively summarize the color characteristics of the road-building facade.

From the perspective of color classification, this article classifies and arranges the main and auxiliary colors according to color types to find out the combination rules of architectural colors. The results show that the color of Macau's World Cultural Heritage Street buildings is characterized by clear hue type, low overall saturation (S = 0–0.6), and medium-high value (V = 0.2–0.8). At the same time, the number of buildings identified as yellow is 33.07%, followed by gray at 31.43%, green at 17.15%, blue at 15.89%, and red at 2.79%.

From the perspective of horizontal space of color, the article summarizes the relationship between architectural style and color sequence. Buildings of artistic value, including baroque municipal offices (Instituto Para Os Assuntos Municipais), the post office of the Macao special administrative and Banco Nacional Ultramarino, 8.7%; Portuguese classical historical buildings accounted for 42.3%, mainly in the warm color of yellow and green; Buildings with Chinese Lingnan style accounted for 11.2%, mainly gray; Modern buildings, moreover, are 35.6 percent blue. Architectural types in different periods on the same road can not only blend in style but also maintain their color characteristics and coordinate with each other in color.

From the perspective of vertical space of color, GH software is used to sort out the color horizon level of “space color” and the change in the color space level. The overall color level perception degree of street building facade space color is affected by distance, street width, and building height. Largo do Senado is taken as the turning point to present the distinctive spatial layers of the “High-rise Road section” and “Macau Varanda Building Road Section.” Space color is greatly influenced by the distance between the two directions of the street and the height of the building at the end of the road.

In conclusion, based on the characteristics of human vision, a new method of color quantization combining color cards and computer vision analysis can be used to comprehensively analyze and comb the street facade colors of Avenida de Almeida Ribeiro in Macao. This method can reduce the error of traditional empirical visual recognition and has ease of use and universality in practice. The conclusion can provide a reference for color planning and facade color restoration of Macao, and has reference significance for the color selection of urban renewal. Nevertheless, the accuracy of this color recognition method needs to be further improved. In future research, the computer vision color perception analysis method used in this article will continue to be enhanced in a more accurate and intelligent direction, providing new ideas for the study of urban color.

## Data availability statement

The original contributions presented in the study are included in the article/supplementary material, further inquiries can be directed to the corresponding author/s.

## Ethics statement

This study was reviewed and approved by the Institutional Research Committee of Macau University of Science and Technology.

## Author contributions

Conceptualization: LY, CZ, and QL. Methodology, formal analysis, data curation, writing—original draft preparation, visualization, and project administration: LY. Software: LY, QL, and YZ. Validation: QL and YZ. Investigation and writing—review and editing: LY and QL. Resources: LY and CZ. Supervision: CZ. All authors have read and agreed to the published version of the manuscript.

## Conflict of interest

The authors declare that the research was conducted in the absence of any commercial or financial relationships that could be construed as a potential conflict of interest.

## Publisher's note

All claims expressed in this article are solely those of the authors and do not necessarily represent those of their affiliated organizations, or those of the publisher, the editors and the reviewers. Any product that may be evaluated in this article, or claim that may be made by its manufacturer, is not guaranteed or endorsed by the publisher.
